# Interleukin-17F Has Anti-Tumor Effects in Oral Tongue Cancer

**DOI:** 10.3390/cancers11050650

**Published:** 2019-05-11

**Authors:** Rabeia Almahmoudi, Abdelhakim Salem, Sakhr Murshid, Mauricio Rocha Dourado, Ehsanul Hoque Apu, Tuula Salo, Ahmed Al-Samadi

**Affiliations:** 1 Department of Oral and Maxillofacial Diseases, University of Helsinki, Biomedicum Helsinki 1, 00014 Helsinki, Finland; abdelhakim.salem@helsinki.fi (A.S.); sakhr.al-kubati@helsinki.fi (S.M.); tuula.salo@helsinki.fi (T.S.); ahmed.al-samadi@helsinki.fi (A.A.-S.); 2 Translational Immunology Research Program (TRIMM), University of Helsinki, Biomedicum Helsinki 1, 00014 Helsinki, Finland; 3 Department of Oral Diagnosis, Piracicaba Dental School, University of Campinas, Campinas-SP, Piracicaba, São Paulo 13083-970, Brazil; mauricio_mrd@hotmail.com; 4 Department of Biomedical Engineering, Institute for Quantitative Health Science and Engineering, Michigan State University, East Lansing, MI 48824, USA; ehsanul.hoqueapu@oulu.fi; 5 Department of Diagnostics and Oral Medicine, Research Group of Cancer Research and Translational Medicine, Medical Faculty, University of Oulu, 90014 Oulu, Finland; 6 Medical Research Centre, Oulu University Hospital, 90220 Oulu, Finland; 7 Cancer and Translational Medicine Research Unit, University of Oulu, 90014 Oulu, Finland

**Keywords:** interleukin-17F, cancer immunotherapy, cytokine therapy, tongue cancer, oral squamous cell carcinoma, proliferation, invasion, apoptosis, angiogenesis, HUVEC cells

## Abstract

We recently showed that extracellular interleukin-17F (IL-17F) correlates with better disease-specific survival in oral tongue squamous cell carcinoma (OTSCC) patients. However, the underlying mechanisms of such effect remain obscure. Here, we used qRT-PCR to assess the expression of IL-17F and its receptors (IL-17RA and IL-17RC) in two OTSCC cell lines (HSC-3 and SCC-25) and in normal human oral keratinocytes (HOKs). IL-17F effects on cancer cell proliferation, migration, and invasion were studied using a live-imaging IncuCyte system, and a Caspase-3/7 reagent was used for testing apoptosis. 3D tumor spheroids were utilized to assess the impact of IL-17F on invasion with or without cancer-associated fibroblasts (CAFs). Tube-formation assays were used to examine the effects of IL-17F on angiogenesis using human umbilical vein endothelial cells (HUVEC). OTSCC cells express low levels of IL-17F, IL-17RA, and IL-17RC mRNA compared with HOKs. IL-17F inhibited cell proliferation and random migration of highly invasive HSC-3 cells. CAFs promoted OTSCC invasion in tumor spheroids, whereas IL-17F eliminated such effect. IL-17F suppressed HUVEC tube formation in a dose-dependent manner. Collectively, we suggest that IL-17F counteracts the pro-tumorigenic activity in OTSCC. Due to its downregulation in tumor cells and inhibitory activity in in vitro cancer models, targeting IL-17F or its regulatory pathways could lead to promising immunotherapeutic strategies against OTSCC.

## 1. Introduction

Oral tongue squamous cell carcinoma (OTSCC) is one of the most prevalent cancers in the oral cavity and has exhibited increased incidence in recent decades [1,2,3]. Unfortunately, OTSCC is associated with a relatively poor prognosis and low 5-year survival rates [2,3,4,5]. Distant metastasis and tumor recurrence remain the key challenges in the current management of OTSCC patients, and thus uncovering and targeting the molecular mechanisms that govern such processes can substantially improve patient survival and treatment outcomes [6].

The ability of cancer cells to migrate and invade host tissue is a major requisite for metastasis [7]. The subsequent events of the metastatic process are complex and include, in addition to cancer cell migration, the intravasation of these cells into the vascular system, extravasation into distant tissues, and metastatic malignant colony formation [8,9]. Therefore, to understand and halt metastasis in the clinical setting, significant research has been devoted to the potential mediators in the tumor microenvironment (TME) via which cancer cells proliferate, migrate, and invade tissues [10]. Recently, cytokine-based immunotherapy has emerged as a novel and attractive therapeutic approach that targets tumorigenic activity in skin cancers (such as melanoma). This approach also holds promise for OTSCC patients [11,12].

The interleukin 17 (IL-17) family consists of six potent proinflammatory cytokines (namely, IL-17A, IL-17B, IL-17C, IL-17D, IL-17E (or IL-25), and IL-17F). These cytokines are secreted by several cell types, such as CD4+ T lymphocytes, mast cells (MCs), and natural killer cells [13,14]. IL-17F, the newest member of the IL-17 family that shares the greatest homology with IL-17A, was reported to provide protective effects against several types of cancer, such as oral, colon, and hepatocellular carcinoma [15,16,17,18,19]. It is believed that this anti-tumor effect is mediated via different and complex mechanisms, including influencing cell cycle arrest and inhibition of angiogenesis [15,17,20]. Furthermore, we showed in our recent multicenter study that MC-derived extracellular (rather than intracellular) IL-17F at the cancer invasion front correlates with better disease-specific survival in OTSCC patients [19]. However, the mechanisms through which IL-17F imparts such favorable effects in OTSCC remain unknown. Therefore, we investigated the functional role of IL-17F in the proliferation, migration, and invasion of OTSCC cell lines. We further tested its effects on blood vessel formation in vitro using human endothelial cell lines.

## 2. Results

### 2.1. Expression of IL-17F and Its Receptors in OTSCC Cells

**Figure 1:**
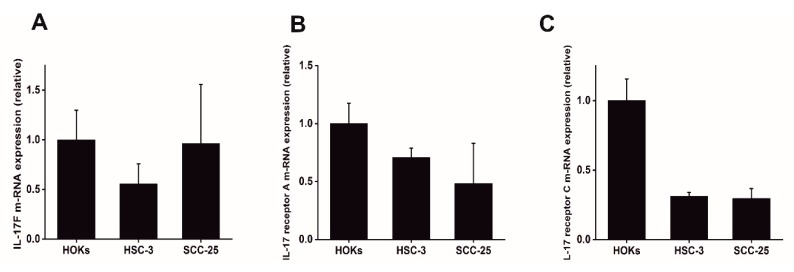
Expression of IL-17F and its receptors in oral tongue squamous cell carcinoma (OTSCC) cells. (**A**) Compared with normal human oral keratinocytes (HOKs), IL-17F mRNA was downregulated in highly invasive HSC-3 cells. (**B**) IL-17F receptor A mRNA was highly decreased in OTSCC cell lines compared to normal controls. (**C**) IL-17F receptor C mRNA was highly decreased in OTSCC cell lines compared to normal controls. Data are presented as means ± standard deviations.

To determine whether IL-17F and its receptors (IL-17RA and IL-17RC) are differentially expressed in OTSCC cells compared to normal oral epithelial control cells, we examined their relative transcript expression in cultured human oral keratinocytes (HOKs) and two OTSCC cell lines, SCC-25 and HSC-3. We found that IL-17F mRNA was expressed at low levels in the most invasive HSC-3 compared with normal HOKs (Figure 1A). Interestingly, the mRNA of the IL-17F receptors (IL-17RA and IL-17RC) were decreased in both HSC-3 and SCC-25 tongue cancer cell lines compared to normal HOKs (Figure 1B,C).

### 2.2. The Effect of IL-17F and IL-17A on OTSCC Cell Proliferation

**Figure 2:**
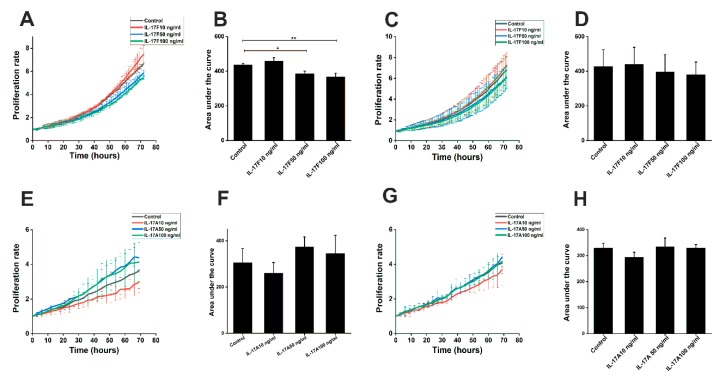
Effects of IL-17F and IL-17A on cancer cell proliferation in the label-free proliferation assay. (**A**,**B**) IL-17F suppresses HSC-3 cell proliferation at concentrations of 50 (*p* = 0.04) and 100 ng/mL (*p* = 0.008) compared with non-stimulated control samples. (**C**,**D**) Treatment of SCC-25 cells with IL-17F shows a similar but non-significant difference. (**E**,**F**) IL-17A produced an opposite effect to IL-17F and showed a slight induction of HSC-3 cell lines. (**G**,**H**) Similarly, IL-17A slightly induced SCC-25 proliferation, although no statistical significance was reached. Data are presented as means ± standard deviations. * *p* ≤ 0.05, ** *p* ≤ 0.01.

To determine whether IL-17F influences cancer cell proliferation, we conducted a label-free proliferation assay on HSC-3 and SCC-25 cells. Our data showed that IL-17F significantly inhibited the proliferation of HSC-3 cells at concentrations of 50 and 100 ng/mL compared with controls (*p* = 0.04 and *p* = 0.008, respectively; Figure 2A,B). Similar trends were seen in the low-invasive SCC-25 cells, however, the differences were not significant (Figure 2C,D). Since an opposite effect has been reported for IL-17A (which shares the highest homology with IL-17F) on cancer cells [20], we also investigated the influence of this cytokine on OTSCC cell proliferation. As expected, IL-17A yielded the opposite effect to IL-17F and slightly induced the cell proliferation of both HSC-3 (Figure 2E,F) and SCC-25 (Figure 2G,H), although this did not reach statistical significance.

### 2.3. The Effect of IL-17F on OTSCC Cell Apoptosis

**Figure 3:**
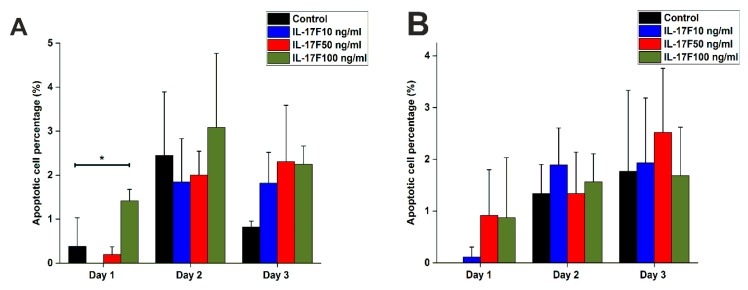
Effects of IL-17F on apoptosis levels in OTSCC cells. (**A**) In Day 1, 100 ng/mL of IL-17F increased apoptosis levels in the highly invasive HSC-3 cell line compared with the non-stimulated controls. A similar trend was also observed in the following days, but the differences were not statistically significant. (**B**) In Day 1, IL-17F (at 50 and 100 ng/mL) seems to slightly increase apoptosis signals in the less-invasive SCC-25 cell line, however, the response was generally less consistent and not statistically significant. In Days 2 and 3, this effect was not found. * *p* ≤ 0.05.

Since we showed that IL-17F inhibited the proliferative capacity of OTSCC cells, we further assessed the potential mechanism behind this observation. Thus, we tested the effect of IL-17F on apoptosis levels in OTSCC cells. As expected, IL-17F induced more apoptosis in OTSCC cells, particularly in HSC-3 at the Day 1 interval (concentration: 100 ng/mL; *p* = 0.04; Figure 3A). A similar trend was observed at Days 2 and 3, however, the differences were not statistically significant. Such effect was not found in SCC-25 cells (Figure 3B).

### 2.4. IL-17F Reduced the Random Migration of the Highly Invasive HSC-3 Cells

**Figure 4:**
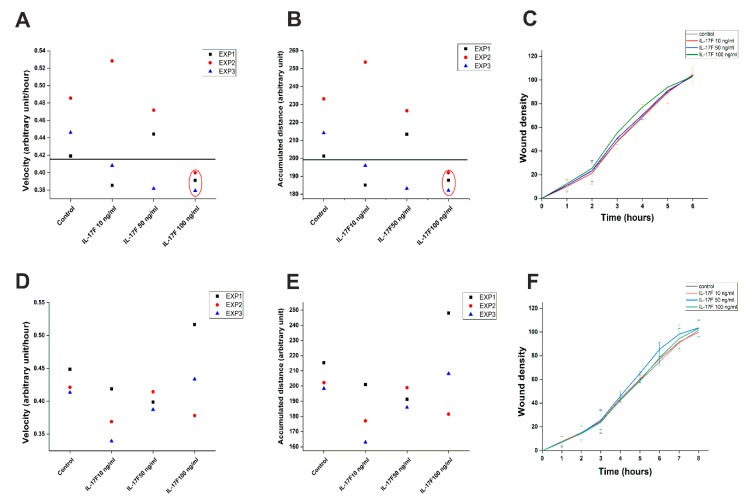
IL-17F reduces random migration in highly invasive HSC-3 cells. (**A**) 100 ng/mL of IL-17F reduced the random migration velocity of the highly invasive HSC-3 cell line compared with the non-stimulated controls. (**B**) 100 ng/mL of IL-17F reduced the accumulated distance of the highly invasive HSC-3 cell line compared with the non-stimulated controls. (**C**) IL-17F had no observable effect on the directed migration (wound density) of HSC-3 cells. (**D**–**F**) IL-17F did not affect the random (velocity and accumulated distance) or directed migration (wound density) of low-invasive SCC-25 cells. Data are presented as means ± standard deviations.

To elucidate the potential effects of IL-17F on the in vitro random and directed migration of OTSCC cell lines, we treated HSC-3 and SCC-25 cells with IL-17F at the concentrations mentioned above. IL-17F at 100 ng/mL decreased the random migration velocity (Figure 4A) and the accumulated distance (Figure 4B) of the highly invasive HSC-3 cells compared to the non-stimulated controls. However, IL-17F showed no observable effect on HSC-3 directed “wound density” migration (Figure 4C) or on the SCC-25 cell random or directed migration (Figure 4D–F).

### 2.5. Evaluating IL-17F Effects on Cell Invasion in Scratch-Wound Assay

**Figure 5:**
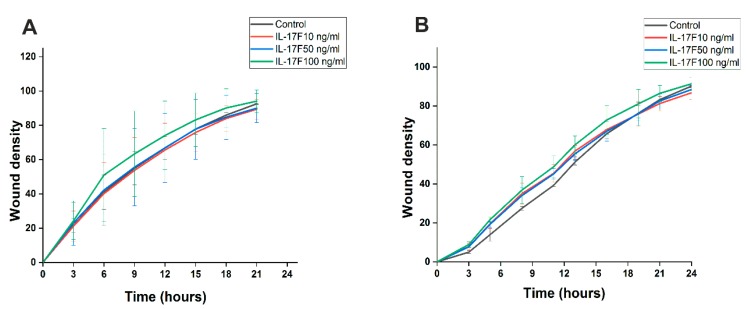
Assessing the effects of IL-17F on cancer cell invasion in a scratch-wound assay. (**A**) HSC-3 cells showed no change in invasiveness when treated with 10, 50, and 100 ng/mL IL-17F for 24 h. (**B**) SCC-25 cells also did not show any observable change in invasiveness when treated with the same concentrations of IL-17F for 24 h. Data are presented as means ± standard deviations.

We next assessed whether IL-17F has an effect on the invasiveness of OTSCC cells in the scratch-wound assay using a Myogel/Collagen matrix. Our data demonstrated that IL-17F had no detectable effect on the invasiveness of HSC-3 (Figure 5A) or SCC-25 cells (Figure 5B).

### 2.6. IL-17F Counteracts HSC-3 Cell Invasion Induced by Cancer-Associated Fibroblasts in Spheroid 3D Model

**Figure 6:**
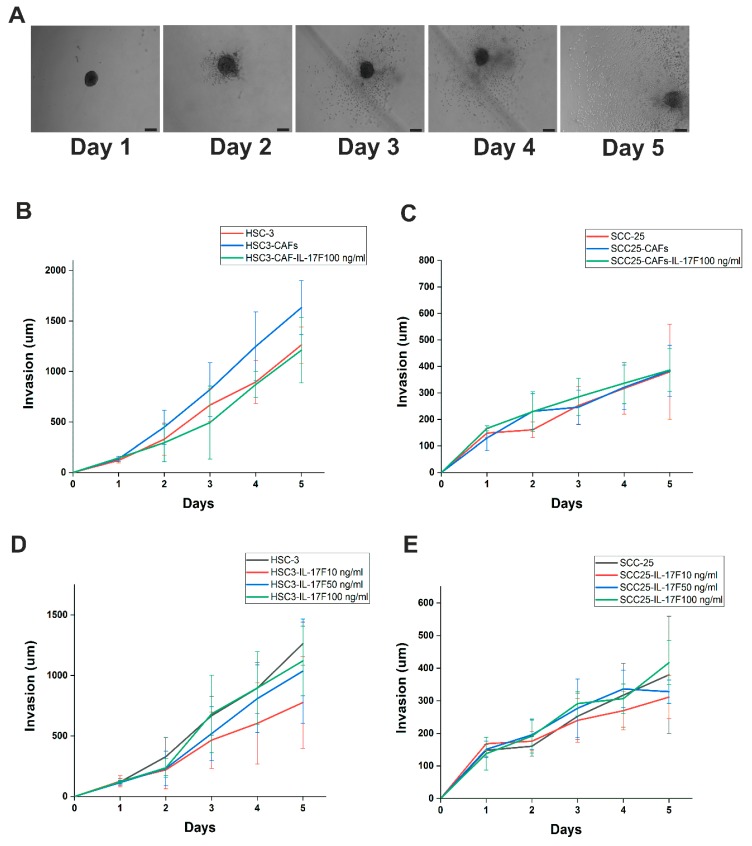
IL-17F prevents the pro-invasive effect of cancer-associated fibroblasts (CAFs) and reduces cancer cell invasion in tumor spheroids. (**A**) Representative figures of HSC-3/CAFs spheroids (Days 1 to 5). Scale = 200 µm. (**B**) The invasion of HSC-3 was enhanced when cells were combined with CAFs (HSC-3/CAFs) rather than with HSC-3 alone. This effect was abolished with addition of 100 ng/mL IL-17F. (**C**) IL-17F had no consistent effect on SCC-25 cell invasion (**D**,**E**) No significant effects of IL-17F on cancer cell invasion were observed when they were cultured without CAFs. Data are presented as means ± standard deviations.

To further delineate the role of IL-17F on OTSCC cell invasion in a 3D spheroid model, HSC-3 or SCC-25 cells alone or in combination with cancer-associated fibroblasts (CAFs) were exposed to various concentrations of IL-17F in a Myogel/Collagen mixture for 5 subsequent days (Figure 6A). As expected, compared to HSC-3 spheroids, cancer cell invasiveness was increased in all three experiments when they were combined with CAFs. This effect was decreased (but not significantly; *p* > 0.05) when 100 ng/mL IL-17F was added (Figure 6B). Other concentrations (10 and 50 ng/mL) did not yield similar effects. No consistent effect was observed in SCC-25 cells (Figure 6C; *p* > 0.05). On the other hand, we did not see any significant effects of IL-17F on the cancer cell invasion when cultured without CAFs (Figure 6D,E).

### 2.7. IL-17F, Unlike IL-17A, Markedly Suppressed Endothelial Cell Tube Formation

**Figure 7:**
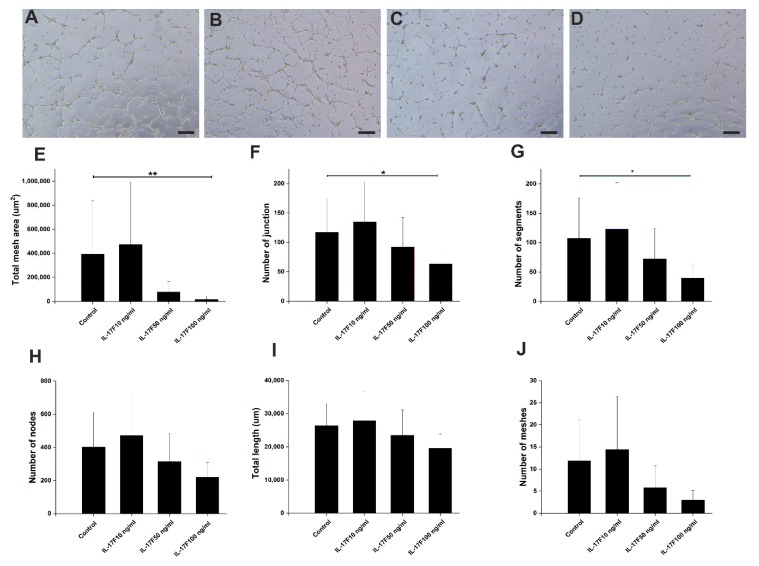
IL-17F inhibits endothelial tube formation in a dose-dependent manner. (**A**) Non-treated human umbilical vein endothelial cells (HUVEC) form clear interlaced tubes when cultured on Matrigel-based matrix. Scale bar = 100 µm. (**B**) 10 ng/mL of IL-17F partially suppressed HUVEC tube formation. (**C**) 50 ng/mL IL-17F induced more inhibition of tube formation. (**D**) A higher dose of IL-17F (100 ng/mL) showed almost complete inhibition of tube formation in HUVEC. (**E**) Morphometric analysis of HUVEC demonstrates that IL-17F reduced the total mesh area (*p* = 0.009, 100 ng/mL). (**F**) IL-17F also reduced the number of junctions (*p* = 0.04, 100 ng/mL). (**G**) IL-17F reduced the number of segments (*p* = 0.02, 100 ng/mL). (**H**) IL-17F reduced the number of nodes. (**I**) IL-17F reduced the total length. (**J**) IL-17F reduced the number of meshes. Data are presented as means ± standard deviations. * *p* ≤ 0.05, ** *p* ≤ 0.01.

We next set up an in vitro human umbilical vein endothelial cell (HUVEC) tube formation assay to directly assess the possible effects of IL-17F on angiogenesis. HUVEC cells formed interlaced tubes in Matrigel^®^ (Figure 7A). IL-17F inhibited HUVEC tube formation in a dose-dependent manner (Figure 7A–D). IL-17F reduced the total mesh area (Figure 7E; *p* = 0.009), number of junctions (Figure 7F; *p* = 0.04), and number of segments (Figure 7G; *p* = 0.02). IL-17F also reduced the number of nodes (Figure 7H), the total length (Figure 7I), and the number of meshes (Figure 7J). We also tested the effects of IL-17A (at the same previous concentrations) on HUVEC tube formation. However, this did not produce any significant changes on the tube formation parameters (Figure S2). In the serum of healthy persons, IL-17F concentration is approximately 400 pg/mL (i.e., 0.4 ng/mL). To test a more physiological concentration, we performed an experiment using 1 ng/mL of both IL-17A and IL-17F. In the majority of tube formation parameters, IL-17F gave significantly lower values compared with IL-17A (Figure S3).

## 3. Discussion

A wide variety of immune and inflammatory cells (such as lymphocytes and MCs) and cytokines constitute a crucial part of the TME that influences cancer cell behavior [21]. Recently, our group reported an interaction between OTSCC cells and immune cells that reduced cancer cell proliferation and invasion area [22]. We also showed that supernatants from activated human MCs were able to downregulate oral oncogenes [23]. Moreover, we found that MC-derived extracellular IL-17F was associated with better overall survival of OTSCC patients [19]. Here, we further demonstrate that IL-17F reduced highly aggressive oral tongue cancer (HSC-3) cell proliferation and random migration. In addition, IL-17F eliminated the CAF-mediated pro-invasive effect on HSC-3. Beyond its influence on cancer cells, IL-17F significantly suppressed HUVEC tube formation.

In our previous study, we reported that cancer cells exhibited negative immunoreactivity to IL-17F in >75% of OTSCC patients. Consistent with this finding, we now demonstrate that OTSCC cell lines (HSC-3 and SCC-25) have rather low mRNA levels of IL-17F. IL-17F conducts its signals via two receptors, IL17RA and IL17RC, which mediate its biological effects in different cells [13]. In this regard, Wright et al. showed that IL-17F binds with approximately 10-fold greater affinity to IL-17RC than to IL-17RA, which is opposite to IL-17A which has greater affinity to IL-17RA [24]. Interestingly, our findings indicate that IL-17F receptors (IL-17RA and IL-17RC) were decreased in both OTSCC cell lines compared with normal oral epithelial cells, and this decrease was more evident in IL-17RC.

The impact of IL-17F on OTSCC cell proliferation is still unknown. IL-17 family cytokines influence, directly and indirectly, the proliferative capacities of certain cell lines such as murine colon and melanoma cancer cell lines. Therefore, Th17 cells and IL-17F have been suggested as promising therapeutic tools in cancer patients [25,26,27]. IL-17A, which shares the greatest homology with IL-17F, induces apoptosis in distinct cell lines such as oligodendrocytes and neural stem cells when added to culture [28,29]. However, it was also recently reported that IL-17A induces the proliferation of nasopharyngeal carcinoma cell lines [20]. Furthermore, it has been suggested that IL-17A plays a critical role in promoting hyperproliferation in squamous cell carcinomas through the induction of STAT3 and its regulated oncogenic and antiapoptotic gene expression [30]. To elucidate this discrepancy, we studied the effects of IL-17A and IL-17F on OTSCC proliferation. Interestingly, our results demonstrated an inhibitory effect of IL-17F on OTSCC cell proliferation. This was contrary to the influence of IL-17A, which showed a slight induction of cell proliferation. We further assessed the effect of IL-17F on OTSCC apoptosis, and it was interesting that the same dose of IL-17F that inhibited HSC-3 proliferation was also able to increase the apoptotic caspase 3/7 signal, at Day 1, in the same cell line. The exact mechanism behind this effect is not yet clear. However, Tong et al. showed that IL-17F can induce cell cycle arrest at the S/G2 transition phase in human colon cancer cell lines, and hence, such cell cycle manipulation may induce an apoptosis cascade [31,32].

Cell migration is an essential event in cancer progression, during which tumor cells disseminate from the primary tumor and reach the TME matrix and vascular system. We therefore studied the effects of IL-17F on cancer cell migration in vitro. Our findings indicated that IL-17F has an inhibitory effect on the random migration velocity of the aggressive HSC-3 cell line. This effect was cell line-dependent, since it was not observed in the less aggressive SCC-25 cell line. These results should be interpreted carefully, as vertical migration was not affected by IL-17F in both cell lines.

Cell invasion is defined as the ability of cells to penetrate basement membranes, tissue barriers, and various matrices [33]. IL-17F treatment did not affect the invasive capacity of OTSCC cells in the wound-closure assay, which was further confirmed in a 3D Myogel/collagen multicellular spheroid model. CAFs are the most abundant cells in the TME [34], and can modulate cancer cell invasion directly by secreting pro-invasive stimuli and indirectly by remodeling the extracellular collagen matrix [35]. Therefore, we used spheroids of OTSCC cell lines, either alone or together with CAFs, and showed that HSC-3 invasion was enhanced (but not significantly) in HSC-3/CAFs spheroids. Similar to our findings, Glentis et al. observed that CAFs isolated from colon cancer patients significantly promoted cancer cell invasion through the laminin basement membrane [36]. More importantly, we were able to abolish the invasion inductive effect by adding IL-17F. This also suggests that IL-17F is possibly involved in orchestrating interactions between different cell types in the TME. While it is difficult to arrive at a solid conclusion on the mechanism behind this effect based on our results, we suggest that IL-17F may interfere with the CAF–cancer cell interaction, since we did not see a decrease of invasion on the cancer cells alone.

Neoangiogenesis, which is the formation of new tubes from pre-existing blood vasculature, is an essential feature in tumor development. Subsequently, inhibition of neoangiogenesis could arrest or halt tumor progression, and would therefore provide an important strategy for anti-cancer therapy [37]. Here, we showed that IL-17F was able to inhibit HUVEC tube formation in a dose-dependent manner. Our findings support previous observations where the IL-17F-driven inhibition was interpreted as an outcome of inducing the transforming growth factor pathway [38]. In the same line, reductions of angiogenesis by IL-17F were reported in in vivo animal studies of hepatocellular and colon carcinomas [15,17]. IL-17F downregulated important proangiogenic cytokines, including IL-6, IL-8, and vascular endothelial growth factor. This could lead to the inhibition of tumor angiogenesis [17]. As IL-17A was reported to promote angiogenesis [39], we compared our results of IL-17F with IL-17A. However, our data did not show any consistent effect of IL-17A on HUVEC tube formation. Such a difference between IL-17F and IL-17A could be explained by the different affinities to the IL-17RA and IL-17RC between these cytokines [24].

Based on the literature, the physiological level of IL-17F in healthy serum samples is 394.3 ± 96.42 pg/mL, and this is reduced to 169.6 ± 50.58 pg/mL in patients with oral cancer [18]. However, to the best of our knowledge, there is no data concerning IL-17F concentrations in tissue. We assume that it is higher than in serum since it is produced by stromal cells, such as MCs [13,19]. Based on this assumption, we tested the effect of low-level (1 ng/mL) IL-17A and IL-17F on HUVEC cell angiogenesis. Even with this concentration, the results had the same trends as the higher test concentrations.

There has been an increasingly growing interest over the past decades in harnessing cytokines for the treatment of cancer. Recently, cytokines have been an attractive target in cancer therapeutics research [11,12]. Preclinical experiments with several murine cancer models showed very promising antitumor effects of granulocyte–macrophage colony-stimulating factor (GM-CSF), IL-2, IL-12, IL-15, and IL-21 [40]. Moreover, IL-2, for instance, has long been approved for the treatment of metastatic renal cell cancer and melanoma [40]. Medications targeting IL-17A showed efficacy in psoriasis and arthritis, which implies that translating these in vitro findings into future clinical use is feasible [41]. The presented anticancer properties of IL-17F could pave the way for novel therapeutic targets in OTSCC patients.

## 4. Materials and Methods

### 4.1. Cell Lines and Culture

We used two different types of human OTSCC cell lines, cancer-associated fibroblasts (CAFs), primary human oral keratinocytes (HOKs), and human umbilical vein endothelial cells (HUVEC). For the OTSCC cell lines, the highly-invasive HSC-3 cell line and the less-invasive SCC-25 cell line were cultured in Dulbecco’s modified Eagle’s medium (DMEM)-12 (Gibco, Paisley, UK). Cultured media was supplied with 10% heat-inactivated fetal bovine serum (FBS), 100 U/mL penicillin, 100 μg/mL streptomycin, 250 ng/mL fungizone, 50 μg/mL ascorbic acid, and 0.1% hydrocortisone. HOKs were cultured in an oral keratinocyte medium supplemented with oral keratinocyte growth supplement and 500 unit/mL of penicillin/streptomycin solution. CAFs were established from tissue explants as described previously [42]. CAFs were cultured in DMEM supplemented with 10% FBS, 100 U/mL penicillin, 100 μg/mL streptomycin, 250 ng/mL fungizone, 1% sodium pyruvate, and 50 μg/mL ascorbic acid. HUVECs (Thermo Fisher Scientific, Waltham, MA, USA) were cultured in 75 cm^2^ tissue-culture flasks using 200PRF medium (Thermo Fisher Scientific) supplemented with low serum growth supplement (LSGS; Thermo Fisher Scientific).

### 4.2. Quantitative Real-Time PCR

Total RNA was purified from HOKs, and HSC-3 and SCC-25 cell lines by using RNeasy Mini-Kit (Qiagen, Düsseldorf, Germany). Two hundred nanograms of pure RNA were used for cDNA synthesis using an iScript cDNA synthesis kit (Bio-Rad, Hercules, CA, USA). For PCR, 10 μL iQ SYBR green, 7 μL water, and 1 μL of 250 nM primer were added to 2 μL of first-strand cDNA. GAPDH was used as the housekeeping gene. Primer sequences are listed in Table S1.

### 4.3. Label-Free Proliferation and Random Migration Assay

The wells of a 96-well plate were coated with 300 μg/mL Myogel [43] and incubated overnight at 37 °C. HSC-3 and SCC-25 cells were seeded at a density of 2 × 10^3^ cells/well and left to adhere overnight. The next day, the old media was replaced with 100 μL of fresh media with or without IL-17F or IL-17A (at 10, 50, or 100 ng/mL). The plate was placed in IncuCyte (Essen Bioscience, Ann Arbor, MI, USA) where images were taken hourly for 3 days. Images were used to measure the proliferation rate (by IncuCyte software, Essen Bioscience) and migration velocity of cancer cells, and their accumulated distance.

### 4.4. IncuCyte™ Caspase-3/7 Apoptosis Assay

The OTSCC cells were cultured in wells of a 96-well plate (at a density of 1 × 10^3^ cells/well) at 37 °C. The next day, the old media was replaced with 100 μL of fresh media with or without IL-17F (at 10, 50, or 100 ng/mL) in addition to the IncuCyte^™^ Caspase-3/7 Reagent at a concentration of 0.5 µM (Essen Bioscience). Images were taken from the center of the wells at a magnification of 10× once a day for 3 subsequent days using EVOS XL Core Cell Imaging System (Thermo Fisher), and the percentages of apoptotic OTSCC cells were calculated.

### 4.5. Scratch-Wound Cell Migration and Invasion Assays

Myogel at a concentration of 300 μg/mL was used to coat the wells of a 96-well Image-lock plate, (Essen Bioscience) which was incubated overnight at 37 °C. The next day, after removing the excess Myogel, HSC-3 and SCC-25 cells were seeded at a density of 30 × 10^3^ cells/well and left to adhere overnight. We used a WoundMaker™ tool (Essen Bioscience) to create homogeneous scratch wounds in each well. After the wounds were made, for the migration assay, the media was replaced with 100 μL of fresh media with or without IL-17F at 10, 50, or 100 ng/mL. For the invasion assay, the wounds were filled with 50 µL of Myogel (2.4 mg/mL)/type 1 rat-tail collagen (0.8 mg/mL). NaOH was used to control the pH of the gel. The gel was allowed to solidify for 30 min before adding 100 µL of fresh media. Both gel and media were prepared with and without IL-17F at 10, 50, or 100 ng/mL. IncuCyte Live-Cell Imaging System (Essen Bioscience) was used to monitor the wound confluence, and images were taken each hour for 24 h (Video S1).

### 4.6. Generation of Tumor Spheroids

The use of spheroids as 3D in vitro models of tumor invasion has the advantage of reconstructing the tumor cellular microenvironments with high reproducibility and applicability [44]. To generate tumor spheroids of 300 to 500 µm diameter, cells (SCC-25 or HSC-3 with or without CAFs) were subcultured and re-suspended in a complete growth medium at a cell density of 1 × 10^4^ cells/mL for HSC-3 and SCC-25 and 5 × 10^3^ cells/mL for CAF. Cell suspensions were then dispensed at 200 µL/well into ultra-low attachment (ULA) 96-well round bottom plates (Corning, NYC, NY, USA) and incubated at 37 °C. Four days later, spheroid formation was confirmed under an eclipse TS100 inverted microscope (Nikon DS-Fi2 camera; Nikon, Tokyo, Japan).

### 4.7. Tumor Spheroid Invasion Assay

Myogel was thawed and diluted to 0.5 mg/mL and mixed with 0.5 mg/mL type 1 rat-tail Collagen (Corning, NYC, NY, USA). One hundred µL of media was removed, and 100 µL Myogel/Collagen1 mixture was gently dispensed into the inner wall of each well of the ULA-plate containing 4-day old spheroids. The gel was allowed to solidify for 30 min, and then another 100 µL of fresh medium was added. Similar to the invasion assay, gel and media were prepared with and without IL-17F at 10, 50, or 100 ng/mL. HSC-3 or SCC-25 alone or in combination with CAFs were exposed to various concentrations of IL-17F in the Myogel/Collagen mixture for 5 subsequent days. Spheroid images were taken using an eclipse TS100 inverted microscope (Nikon DS-Fi2 camera; Nikon, Tokyo, Japan). Cancer cell invasion was calculated as the distance from the farthest cancer cell to the center of the tumor spheroid.

### 4.8. Tube Formation Assay

We added 100 µL of Matrigel^®^ (Corning, NYC, NY, USA) to a 24-well plate and left it for 30 min at 37 °C. HUVEC were seeded at a density of 8 × 10^4^ cells/well, and media with and without IL-17F or IL-17A at concentrations of 1, 10, 50, or 100 ng/mL was added. For the positive inducer and control samples, cells were diluted in low serum growth supplement (LSGS)-supplemented 200PRF medium, which contains fibroblast growth factor (3 ng/mL). Cells were then transferred into the Matrigel^®^-coated wells and incubated for 6 h at 37 °C. Images were taken using an eclipse TS100 inverted microscope (Nikon DS-Fi2 camera).

### 4.9. ImageJ Software Analysis

We used ImageJ software (Wayne Rasband, National Institute of Health, Bethesda, MD, USA) to analyze the images obtained from migration, spheroids, and angiogenesis assays. Three cells were selected from each well, and their movements were tracked for 8 h (Video S2). The Chemotaxis and Migration Tool (Ibidi, Martinsried, Germany) was used to measure random cells’ velocities and accumulated distances. For tube formation analysis, ImageJ software was applied to measure several parameters (Figure S1), including: (1) Nodes (represented as pixels with three neighbors as a circular dot, (2) Junctions (corresponding to nodes or a group of fusing nodes), (3) Segments (elements delimited by two junctions), and (4) Meshes (areas enclosed by segments or master segments).

### 4.10. Statistical Analysis

All experiments were repeated independently three to four times, each in duplicate or triplicate. Values are given as means ± standard deviations. Data were analyzed using IBM SPSS Statistics version 24.0 (IBM Corp., Armonk, NY, USA). To determine the statistical significance, we performed non-parametric Kruskal–Wallis and Friedman tests. Statistical significance was set to *p* < 0.05.

## 5. Conclusions

Our data, taken together, suggest promising anti-tumor effects of IL-17F on OTSCC by inhibiting cancer cell proliferation, invasion, and endothelial angiogenesis. Indeed, employing cytokines in immunotherapy has recently been an attractive area in cancer research [11,12]. The success of IL-17A-based medications against other immune disorders (e.g., psoriasis and arthritis) implies that translating these in vitro findings into future therapies is feasible [41]. Lastly, such emerging anticancer evidence of IL-17F necessitates more functional studies to further understand the intricate mechanisms behind its role in OTSCC.

## Supplementary Materials

The following are available online at https://www.mdpi.com/2072-6694/11/5/650/s1, Figure S1: Morphometric analysis of endothelial tube formation assay, Figure S2: IL-17A effect on tube-formation parameters of human umbilical vein endothelial cell, Figure S3: IL-17F and IL17A effects at 1 ng/mL on tube-formation parameters of human umbilical vein endothelial cell, Table S1: Sequences of the used human gene primers, Video S1: Scratch-wound cell migration assay of the HSC-3 cells, Video S2: Cell movement tracking of the HSC-3 cells.
