# Supplementary material for: Interleukin-17F Has Anti-Tumor Effects in Oral Tongue Cancer
Source: Cancers (Basel). 2019 May 11;11(5):650. doi: 10.3390/cancers11050650 (PMC6562684; doi:10.3390/cancers11050650)
Supplement: Supplementary file 1 [file cancers-11-00650-s001.zip › Supplementary Table and Figures.pdf]

Supplementary Table 1. Sequences of the used human gene primers.

Gene	Forward	Reverse
**IL-17F**	5′-CCCTGGAATTACACTGTCACTTGG -3′	5′-GAAAGAAACAGAGCAGCCTTGGTG-3′
**IL-17RA**	5′-CTGGTTCATCACGGGCATCTCC-3′	5′-GGTGGTCGGCTGAGTAGATGATC-3′
**IL-17RC**	5′-CGTCACTGTGGACAAGGTTCTCG-3′	5′-TCGTGGAGGCTTTGCTGGGTAG-3′
**GAPDH**	5′-AAGGTCATCCCTGAGCTG-3′	5′-TGCTGTAGCCAAATTCGTTG-3′

GAPDH: Glyceraldehyde 3-phosphate dehydrogenase; IL-17F: Interleukin-17F; IL-17RA: Interleukin-17 Receptor A; IL-17RC: Interleukin-17 Receptor C.

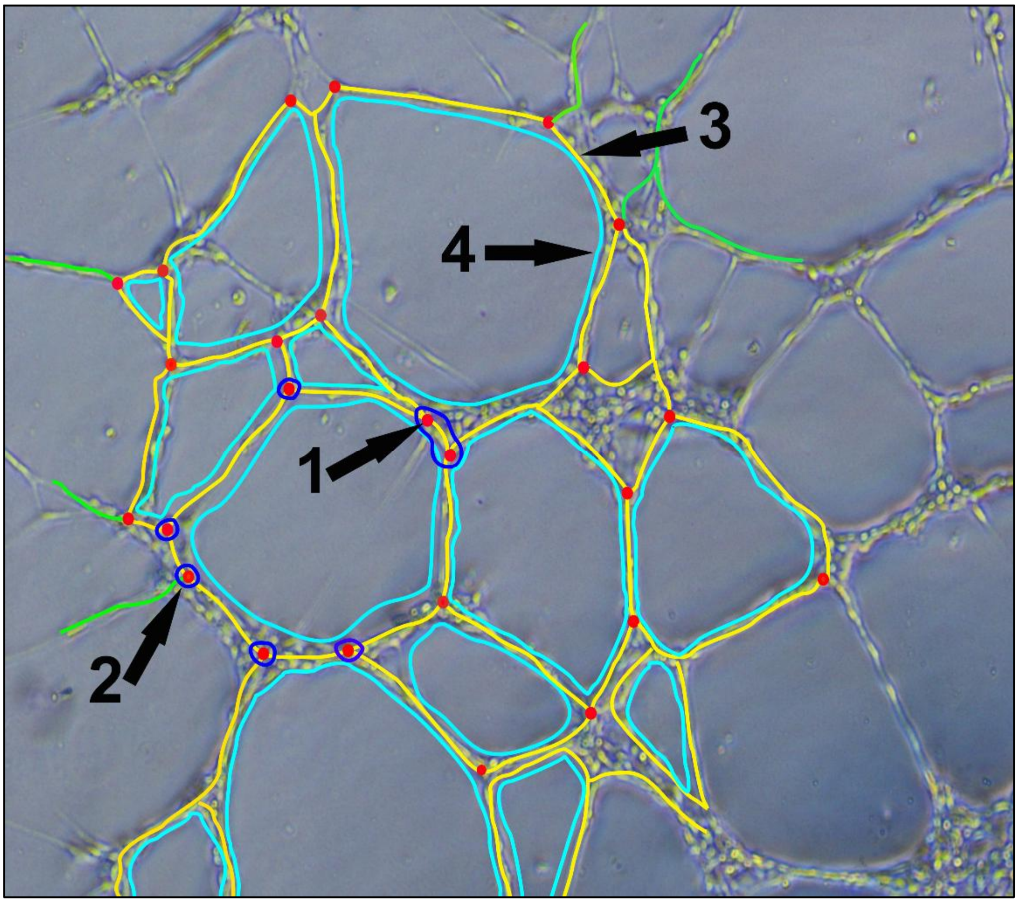

Supplementary Fig. 1 Morphometric analysis of endothelial tube formation assay. Tubes and vascular pattern were analysed using ImageJ software. The following parameters were used for the quantification of the endothelial tube networks: 1) Nodes represent pixels with 3 neighbours as a circular dot; 2) Junctions correspond to nodes or group of fusing nodes; 3) Segments refer to elements delimited by two junctions; 4) Meshes are areas enclosed by segments or master segments.

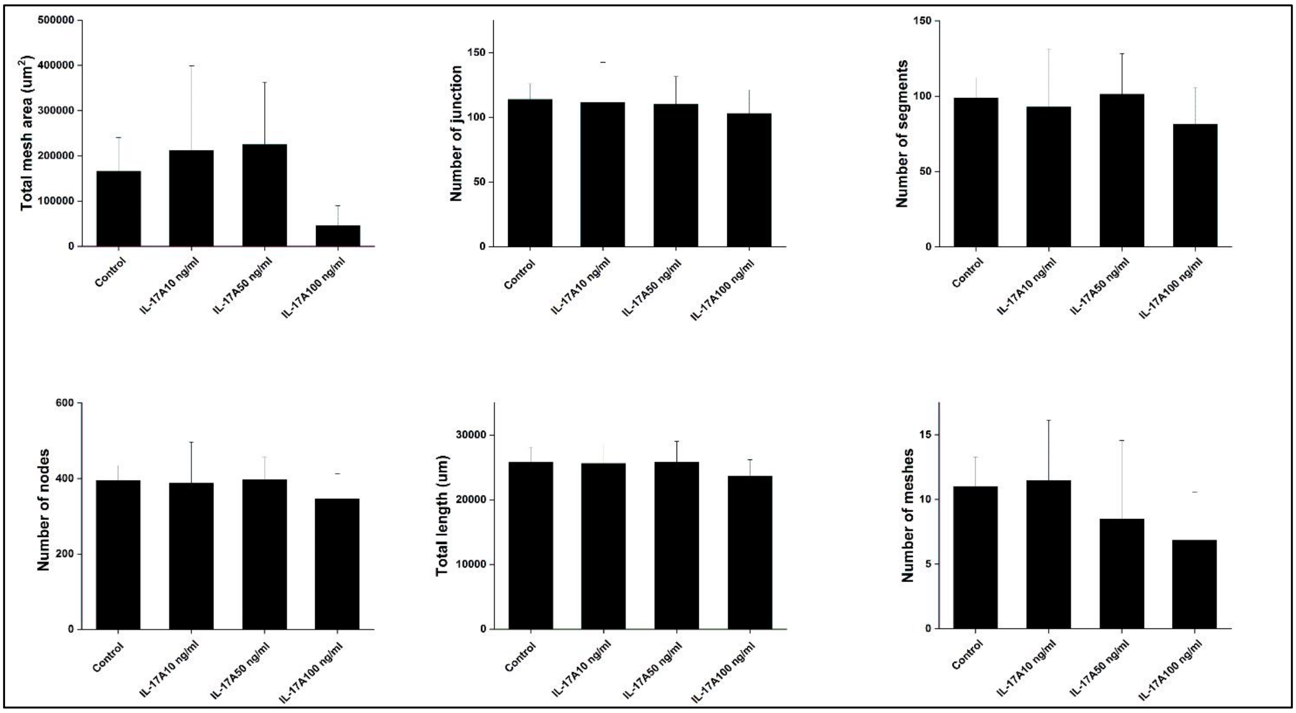

Supplementary Fig. 2 IL-17A did not show a significant effect on tube-formation parameters of human umbilical vein endothelial cells.

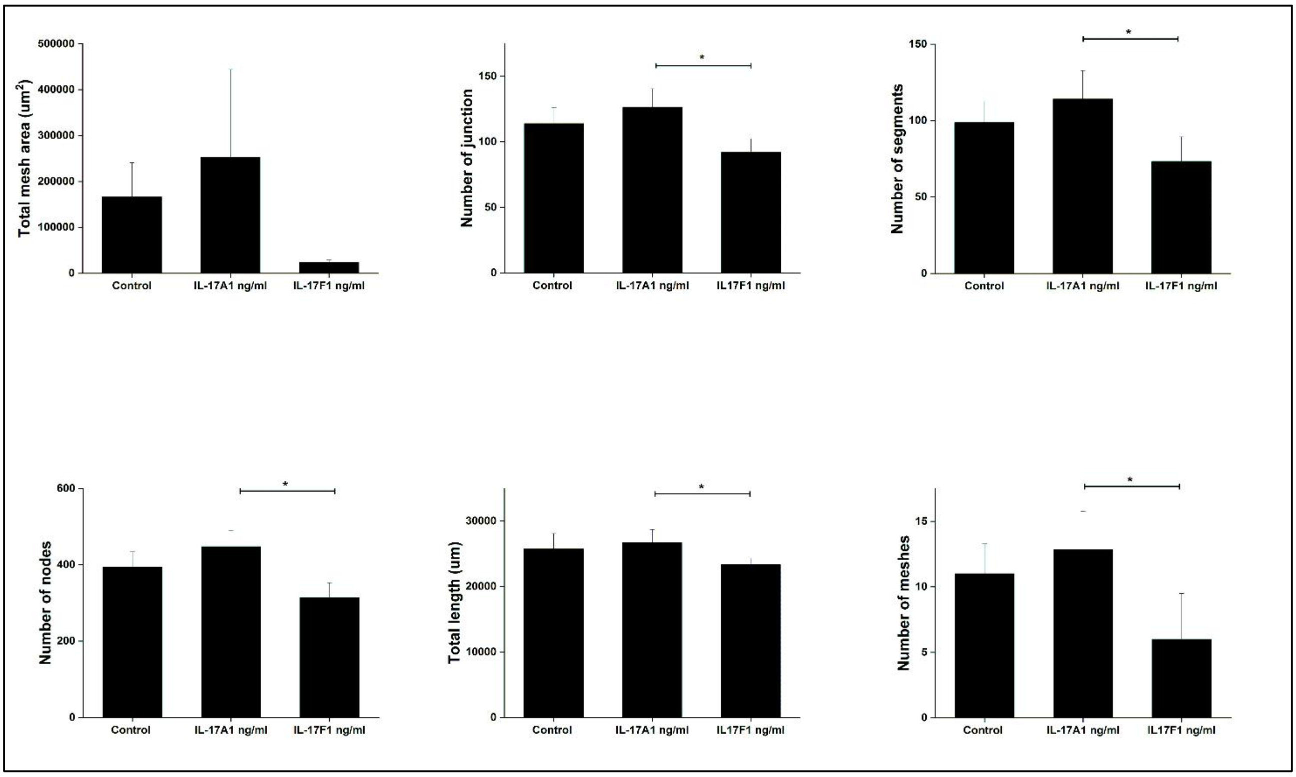

Supplementary Fig. 3 IL-17F at 1 ng/ml induces the tube-formation parameters of human umbilical vein endothelial cells, while IL-17A, at the same concentration, shows an opposite effect.
